# Epidemiology and mortality rate of thyroid storm in Thailand: Analysis using a national in-patient database

**DOI:** 10.1371/journal.pone.0357142

**Published:** 2026-08-27

**Authors:** Jin Sothornwit, Suranut Charoensri, Dueanchonnee Sribenjalak, Chatlert Pongchaiyakul

**Affiliations:** Department of Medicine, Division of Endocrinology and Metabolism, Faculty of Medicine, Khon Kaen University, Khon Kaen, Thailand; Tsinghua University, CHINA

## Abstract

**Objective:**

To investigate the nationwide incidence, temporal trends, of thyroid storm among hospitalized patients with thyrotoxicosis in Thailand, to compare in-hospital mortality between patients with and without thyroid storm, and to identify independent predictors of mortality and healthcare resource utilization among patients with thyroid storm.

**Methods:**

A retrospective population-based study was conducted using the National Health Security Office database (2017–2024), including adult patients hospitalized with thyrotoxicosis recorded as the principal diagnosis. Thyroid storm was identified by ICD-10 code E05.5, while other thyrotoxicosis diagnoses without thyroid storm served as the comparator group. Temporal trends in incidence were assessed by Poisson regression, and multivariable logistic regression models identified independent predictors of mortality and prolonged length of hospital stay among patients with thyroid storm.

**Results:**

Among 4,418 admissions with thyrotoxicosis as the principal diagnosis, 1,160 (26.2%) were thyroid storm and 3,258 (73.8%) were thyrotoxicosis without thyroid storm. The average annual incidence was 0.22 cases per 100,000 person-years, rising from 0.15 (2017) to 0.32 (2024) (p for trend < 0.001). In-hospital mortality was significantly higher in patients with thyroid storm than in those without (18.0% versus 1.0%; p < 0.001). After multivariable adjustment, thyroid storm was independently associated with increased mortality (aOR 11.17; 95% CI 7.41–16.85; p < 0.001). Septic shock (aOR 5.26), cardiogenic shock (aOR 4.90), and acute kidney injury (aOR 3.72) were the strongest independent predictors of death. The median length of hospital stay was 6 days (P25-P75 4–10).

**Conclusions:**

The incidence of thyroid storm in Thailand is rising, with a high in-hospital mortality of 18.0%. Mortality is independently driven by advanced age, male sex, chronic liver disease, and acute multi-organ complications, particularly septic shock, cardiogenic shock, and acute kidney injury. Prolonged hospitalization is largely driven by pneumonia, acute kidney injury, and chronic liver disease. Early recognition and aggressive management of precipitating factors, particularly pneumonia, and expanded insurance coverage for advanced therapies are essential to improve survival.

## Introduction

Thyroid storm represents the most extreme and life-threatening spectrum of thyrotoxicosis, characterized by systemic decompensation and multi-organ failure. [1] Despite significant therapeutic advancements in critical care, thyroid storm remains associated with a formidable mortality rate, ranging from 1.2% to 30% in contemporary clinical series. [1–8] The clinical diagnosis of this condition is predominantly based on phenotypic presentation and standardized scoring systems such as the Burch-Wartofsky Point Scale and the Japan Thyroid Association criteria. [1,6,9] However, the rarity of the condition and the absence of definitive biochemical thresholds necessitate large-scale epidemiological investigations to refine our understanding of its natural history and prognostic determinants. [5]

On a global scale, the incidence and clinical outcomes of thyroid storm exhibit significant geographic variation, with national database studies from Japan, the United States, and Germany reporting incidence rates between 0.2 and 0.7 per 100,000 person-years. [2,6,8] However, data from Southeast Asia, particularly from middle-income countries with universal healthcare frameworks, remain scarce. This study therefore aims to determine the nationwide incidence, evaluate temporal trends in mortality and healthcare utilization, and identify independent predictors of in-hospital death in Thailand over an eight-year period, with the goal of informing regional management guidelines and improving clinical outcomes for this endocrine emergency.

## Materials and methods

### Study design and data source

This retrospective study analyzed inpatient summary data of adult patients (aged ≥18 years) hospitalized with thyrotoxicosis in hospitals within the National Health Security Office (NHSO) network in Thailand between January 2017 and December 2024. The data were accessed for research purposes on 26 January 2026. Because the database contained only de-identified records, the authors did not have access to information that could identify individual participants during or after data collection. The inclusion criteria were adult patients (aged ≥18 years) hospitalized with a principal discharge diagnosis of thyrotoxicosis (including thyroid storm). Admissions were excluded if the patient was younger than 18 years or if thyrotoxicosis was recorded only as a secondary diagnosis without thyroid storm. Clinically, thyrotoxicosis was defined as the syndrome resulting from excess circulating thyroid hormone, and thyroid storm as its life-threatening, decompensated form characterized by thermoregulatory, cardiovascular, hepatic–gastrointestinal, and central nervous system dysfunction. In Thai clinical practice, there is no single nationally mandated diagnostic standard; the clinical diagnosis of thyroid storm is made by the treating physician, most often guided by the Burch–Wartofsky Point Scale (BWPS) and/or the Japan Thyroid Association (JTA) criteria. [1,6,9] Because the NHSO database does not record these underlying clinical scores, thyroid storm and thyrotoxicosis were operationally identified based on the principal-diagnosis ICD-10 codes assigned by the attending physician, rather than being re-adjudicated by the study team.

The dataset provided information on principal and secondary diagnoses (up to 22 diagnosis codes), procedures, discharge status, and length of hospital stay (LOS). Diagnoses and procedures were coded using the International Classification of Diseases, Ninth and Tenth Revisions (ICD-9 and ICD-10). Thyroid storm was operationally identified by the ICD-10 code E05.5 recorded as the principal diagnosis. Other forms of thyrotoxicosis without thyroid storm were identified based on ICD-10 codes E05.0, E05.1, E05.2, E05.3, E05.4, E05.8, E05.9, E06.0, E06.1, E06.2, E06.3, E06.4, E06.5, E06.9, and O90.5 recorded as the principal diagnosis only.

For the thyrotoxicosis without storm group, etiologies were derived directly from the principal diagnosis code. For the thyroid storm group, etiologies were identified from the secondary diagnosis codes; if no specific etiological code was recorded, the case was classified as unspecified thyrotoxicosis. Comorbidities, in-hospital complications, and potential precipitating factors (e.g., pneumonia, sepsis) were identified from up to 22 secondary ICD-10 diagnosis codes recorded for the same admission, and life-sustaining procedures from ICD-9-CM procedure codes. A condition was regarded as a complication or precipitating factor when its code co-occurred within the same admission. The complete list of ICD codes used to define all study variables is provided in S1 Table.

### Main outcome measures

The outcomes of interest included incidence, in-hospital mortality, clinical characteristics, length of hospital stay, and precipitating factors. The incidence of thyroid storm was calculated by dividing the total number of hospital discharges by the annual Thai population estimates provided by the Bureau of Registration Administration. To ensure a conservative national estimate, each hospitalization was analyzed as an independent observation. Patient characteristics included age, sex, etiologies of thyrotoxicosis, comorbidities, and associated life-sustaining procedures.

### Statistical analysis

Continuous variables were expressed as mean and standard deviation (SD) or median (25th-75th percentile, P25-P75), depending on data distribution as assessed by the Kolmogorov-Smirnov test. Categorical variables were presented as numbers and percentages. Comparisons between patients with thyroid storm and those without were performed using the Mann-Whitney U test, Student’s t-test, Chi-square test, or Fisher’s exact test, as appropriate. Temporal trends in the annual hospitalization (incidence) rate were assessed by Poisson regression, with the annual case count as the dependent variable, calendar year modelled as a continuous covariate, and the log of the annual mid-year population as an offset; the p value for trend corresponds to the Wald test of the calendar-year coefficient.

Univariable analyses identified factors associated with in-hospital mortality among patients with thyroid storm. Variables with p < 0.10 in univariable analyses and clinically relevant parameters were candidates for multivariable logistic regression. To avoid over-adjustment bias, variables representing intermediate outcomes in the causal pathway to death (cardiac arrest and ventricular arrhythmia) were excluded from the multivariable model. The number of predictors was limited according to the events-per-variable criterion (minimum 10 events per variable) to minimize overfitting. Multicollinearity was assessed using Variance Inflation Factors (VIF); all VIF values were below 5 (S2 Table). Model discrimination was assessed by the area under the receiver operating characteristic curve (AUC) and calibration by the Hosmer-Lemeshow goodness-of-fit test.

Because length of hospital stay (LOS) data were right-skewed, the variable was summarized using the median (P25–P75) and dichotomized at the median value. Prolonged LOS was defined as a hospital stay longer than the median LOS. Multivariable logistic regression was then performed to identify factors independently associated with prolonged LOS among patients hospitalized with thyroid storm, applying the same events-per-variable criterion (minimum 10 events per variable) to minimize overfitting.

All statistical tests were two-sided, with a p-value less than 0.05 considered significant. Analyses were performed using IBM SPSS Statistics version 29.0.2.0.

### Ethics approval and consent to participate

The study protocol was approved by the Institutional Review Board of Khon Kaen University (IRB No. 00001189, HE691021). The requirement for informed consent was waived due to the retrospective nature of the study. The study was conducted in accordance with the Declaration of Helsinki.

## Results

### Demographic data

**Fig 1:**
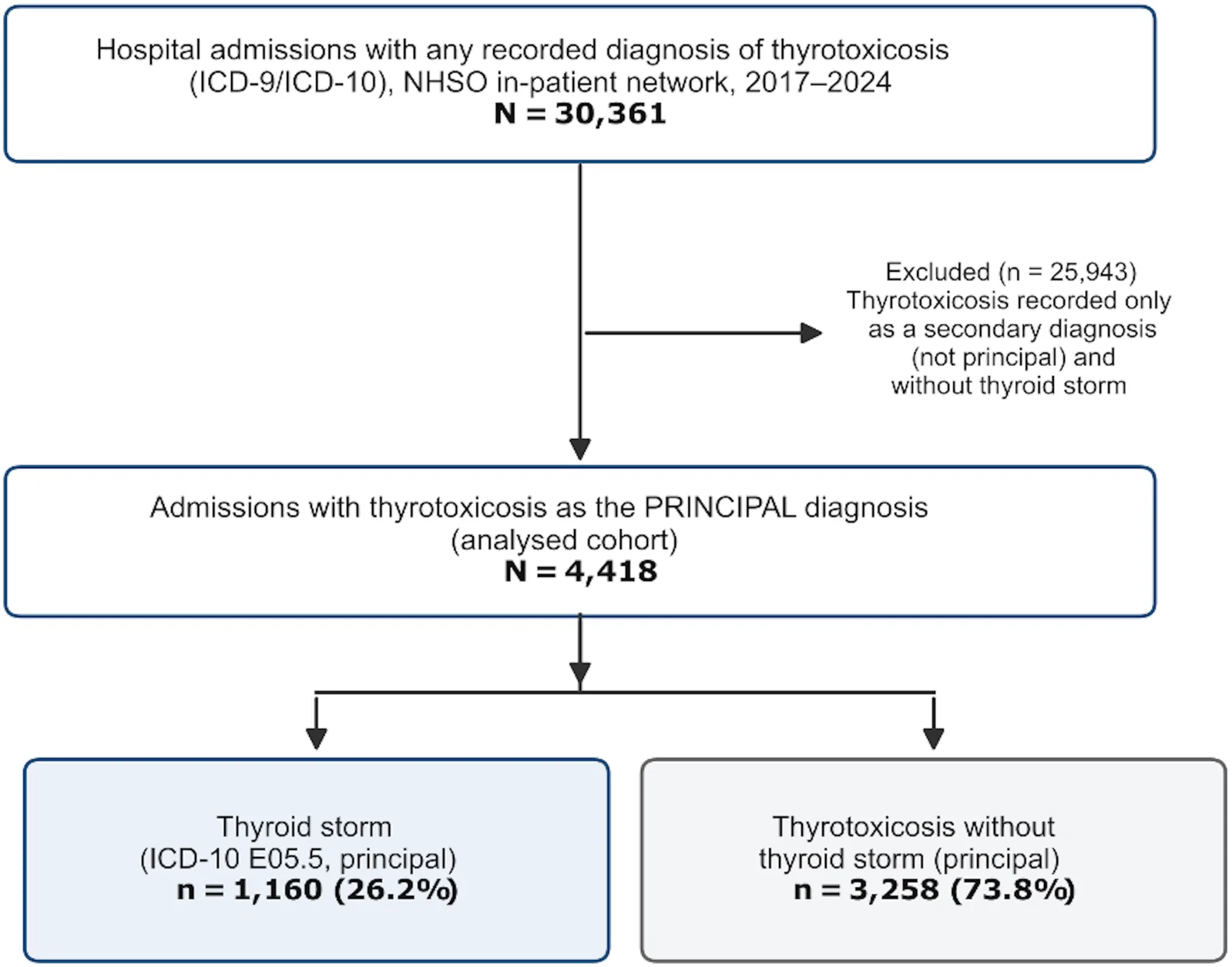
Flow diagram of the study cohort derivation. Of the 30,361 admissions with thyrotoxicosis in the NHSO database (2017–2024), 25,943 admissions with thyrotoxicosis as a secondary diagnosis only were excluded. The final analyzed cohort comprised 4,418 admissions with thyrotoxicosis as the principal diagnosis, consisting of 1,160 (26.2%) thyroid storm cases and 3,258 (73.8%) thyrotoxicosis without thyroid storm cases.

**Fig 2:**
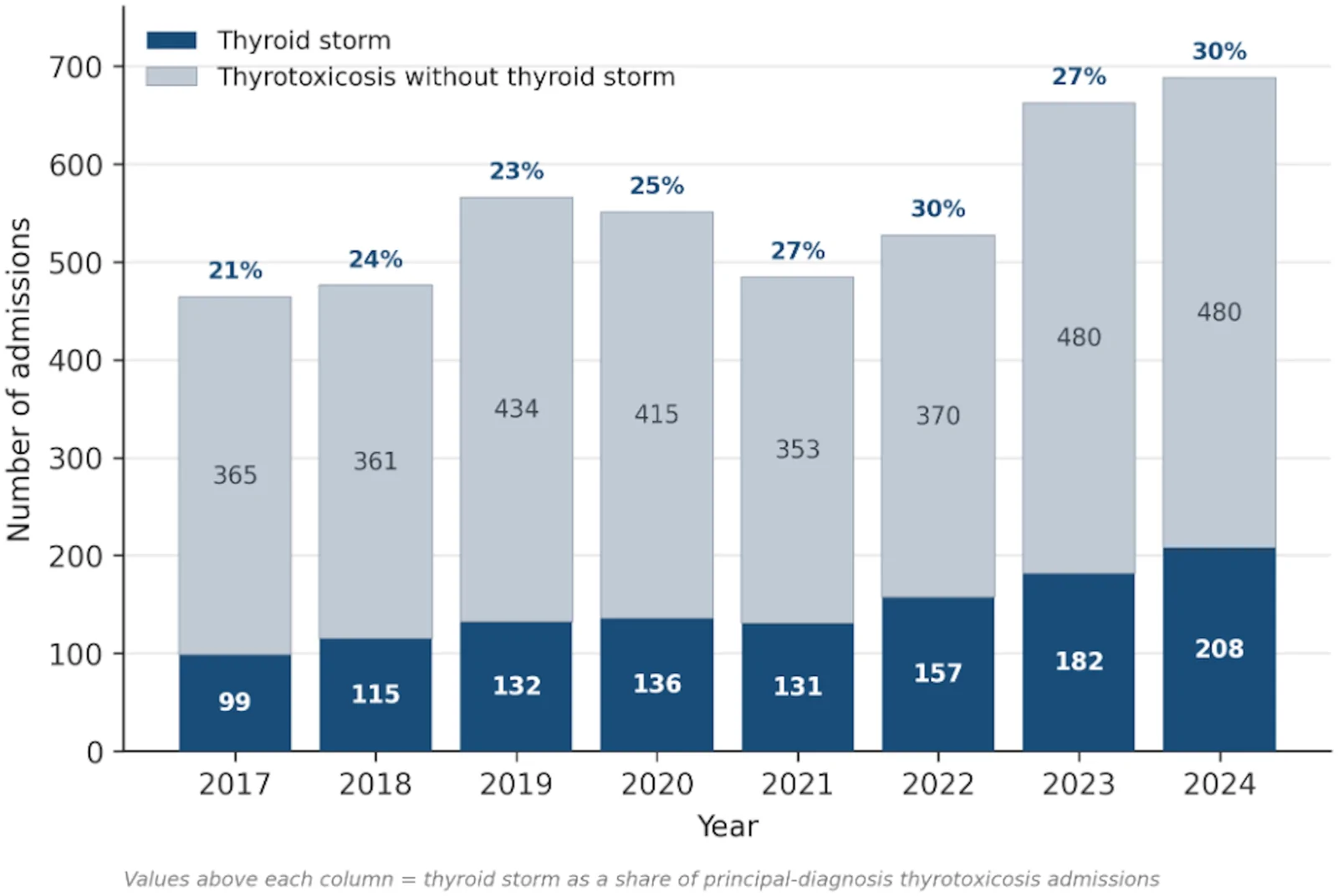
Annual admissions for thyrotoxicosis with and without thyroid storm in Thailand, 2017–2024. Values above each column indicate thyroid storm as a share of all principal-diagnosis thyrotoxicosis admissions. Dark bars represent admissions with thyroid storm, and light bars represent admissions without thyroid storm.

During the study period, 30,361 admissions carried a diagnosis of thyrotoxicosis in any diagnostic field. After excluding 25,943 admissions in which thyrotoxicosis was recorded only as a secondary diagnosis (without thyroid storm), 4,418 admissions with thyrotoxicosis as the principal diagnosis formed the analyzed cohort (Fig 1). Of these, 1,160 (26.2%) were thyroid storm and 3,258 (73.8%) were thyrotoxicosis without thyroid storm (Fig 2).

Between January 2017 and December 2024, the overall annual hospitalization rate for thyroid storm demonstrated a significant and steady upward trend. The rate doubled from 0.15 cases per 100,000 population in 2017 to a peak of 0.32 cases per 100,000 population in 2024. The mean annual incidence over the eight-year study period was 0.22 cases per 100,000 person-years. Poisson regression analysis confirmed a statistically significant temporal trend during the study period (p for trend < 0.001).

### Clinical characteristics

The median age of patients diagnosed with thyroid storm was 49 years (P25-P75, 35–63), which was comparable to the median age of 49 years (P25-P75, 34–61) observed in the thyrotoxicosis without thyroid storm group. Female patients predominated in both cohorts. Regarding etiology, unspecified thyrotoxicosis was identified as the leading cause in both groups. Patients presenting with thyroid storm exhibited a significantly higher burden of chronic comorbidities compared to those without thyroid storm, particularly chronic liver disease, chronic lung disease, and coronary artery disease (all p < 0.05). Detailed comparisons of demographic data and baseline comorbidities are provided in Table 1.

**Table 1:** Patients’ clinical characteristics.

	Thyroid storm (N = 1,160)	Thyrotoxicosis without thyroid storm (N = 3,258)	p-value
Age, median (P25-P75), years	49 (35-63)	49 (34-61)	0.187^a^
Female sex, N (%)	728 (62.8)	2,245 (68.9)	<0.001^b^
Etiologies of thyrotoxicosis, N (%)			
Thyrotoxicosis with diffuse goiter (Graves’ disease)	72 (6.2)	523 (16.1)	<0.001^b, c^
Toxic single thyroid nodule	1 (0.1)	40 (1.2)	
Toxic multinodular goiter	5 (0.4)	217 (6.7)	
Thyrotoxicosis from ectopic thyroid tissue	0 (0.0)	6 (0.2)	
Thyrotoxicosis factitial	0 (0.0)	4 (0.1)	
Other thyrotoxicosis	0 (0.0)	18 (0.6)	
Thyrotoxicosis, unspecified	1,079 (93.0)	2310 (70.9)	
Acute thyroiditis	1 (0.1)	53 (1.6)	
Subacute thyroiditis	0 (0.0)	9 (0.3)	
Chronic thyroiditis with transient thyrotoxicosis	0 (0.0)	1 (0.0)	
Autoimmune thyroiditis	2 (0.2)	46 (1.4)	
Drug-induced thyroiditis	0 (0.0)	1 (0.0)	
Other chronic thyroiditis	0 (0.0)	1 (0.0)	
Thyroiditis, unspecified	0 (0.0)	29 (0.9)	
Comorbidities, N (%)			
Coronary artery disease	29 (2.5)	30 (0.9)	<0.001^b^
Chronic liver disease	110 (9.5)	71 (2.2)	<0.001^b^
Chronic kidney disease	29 (2.5)	83 (2.5)	0.929^b^
Chronic lung disease	38 (3.3)	61 (1.9)	0.006^b^
Diabetes mellitus	149 (12.8)	368 (11.3)	0.159^b^
Pregnancy	19 (1.6)	2 (0.1)	<0.001^b^
Procedures			
Mechanical ventilation	443 (38.2)	115 (3.5)	<0.001^b^
Total thyroidectomy	3 (0.3)	206 (6.3)	<0.001^d^

P25-P75, 25th-75th percentile. ^a^Mann-Whitney U test; ^b^Chi-square test; ^c^Overall comparison; ^d^Fisher’s Exact test

### Comorbidities and procedures in patients with thyroid storm

Among patients hospitalized with thyroid storm between 2017 and 2024, atrial arrhythmia was the most prevalent comorbidity (53.4%), followed by heart failure (40.7%) and pneumonia (17.3%). Other clinical manifestations, such as adrenal insufficiency and altered mental status, were infrequently observed, with prevalences of 1.2% and 2.3%, respectively.

In the univariable analyses, male sex and advanced age were significantly associated with increased in-hospital mortality. Furthermore, mortality rates were markedly higher among patients presenting with a higher burden of comorbidities, infectious complications, septic shock, and cardiogenic shock. Major cardiovascular and neurological complications, as well as the requirement for life-sustaining organ support, specifically mechanical ventilation and hemodialysis, were also identified as significant predictors of a fatal outcome (all p < 0.05; Table 2).

**Table 2:** Baseline comorbidities, procedures, and univariable analysis of in-hospital mortality in patients with thyroid storm.

	Number of patients (%)	In-hospital mortality, N (%)	p-value
Sex			
Male	432 (37.2)	102 (23.6)	<0.001^a^
Female	728 (62.8)	107 (14.7)	
Age group			
18-39	372 (32.1)	43 (11.6)	<0.001^a^
40-64	532 (45.9)	109 (20.5)	
≥65	256 (22.0)	57 (22.3)	
Baseline comorbidities			
Coronary artery disease	29 (2.5)	11 (37.9)	0.005^a^
Chronic liver disease	110 (9.5)	38 (34.5)	<0.001^a^
Chronic kidney disease	29 (2.5)	5 (17.2)	0.912^a^
Chronic lung disease	38 (3.3)	7 (18.4)	0.947^a^
Diabetes mellitus	149 (12.8)	38 (25.5)	0.011^a^
Pregnancy	19 (1.6)	0 (0.0)	0.034^b^
Shock and organ failure			
Septic shock	107 (9.2)	59 (55.1)	<0.001^a^
Cardiogenic shock	41 (3.5)	23 (56.1)	<0.001^a^
Other shock	9 (0.8)	3 (33.3)	0.210^b^
Acute liver failure	30 (2.6)	18 (60.0)	<0.001^a^
Acute kidney injury	166 (14.3)	89 (53.6)	<0.001^a^
Acute infection			
Sepsis	104 (9.0)	28 (26.9)	0.013^a^
Pneumonia	201 (17.3)	69 (34.3)	<0.001^a^
Intestinal infection	44 (3.8)	7 (15.9)	0.711^a^
Acute cystitis	5 (0.4)	1 (20.0)	1.000^b^
Acute pyelonephritis	19 (1.6)	4 (21.1)	0.762^b^
Cardiovascular complications			
Acute coronary syndrome	41 (3.5)	16 (39.0)	<0.001^a^
Heart failure	472 (40.7)	97 (20.6)	0.063^a^
Atrial fibrillation and flutter, supraventricular tachycardia	620 (53.4)	142 (22.9)	<0.001^a^
Ventricular arrhythmia	21 (1.8)	11 (52.4)	0.075^b^
Cardiac arrest	50 (4.3)	38 (76.0)	<0.001^b^
Neurological complications			
Acute intracranial hemorrhage	3 (0.3)	0 (0.0)	1.000^b^
Acute ischemic stroke	82 (7.1)	22 (26.8)	0.031^a^
Altered mental status	27 (2.3)	5 (18.5)	0.945^b^
Other acute complications			
Pulmonary embolism	1 (0.0)	0 (0.0)	1.000^b^
Diabetic ketoacidosis	16 (1.4)	5 (31.3)	0.165^b^
Adrenal insufficiency	14 (1.2)	6 (42.9)	0.015^b^
Disseminated intravascular coagulation	22 (1.9)	12 (54.5)	<0.001^a^
Trauma	48 (4.1)	9 (18.8)	0.893^b^
Non-compliance to medication	0 (0.0)	0 (0.0)	--
Numbers of comorbidities			
0	179 (15.4)	4 (2.2)	<0.001^a, c^
1	225 (19.4)	15 (6.7)	
2	258 (22.2)	36 (14.0)	
≥3	498 (42.9)	154 (30.9)	
Procedures			
Mechanical ventilation	443 (38.2)	173 (39.1)	<0.001^a^
Hemodialysis (HD)	30 (2.6)	16 (53.3)	<0.001^b^
Therapeutic plasma exchange (TPE)	6 (0.5)	2 (33.3)	0.296^b^
Intra-aortic balloon pumping (IABP)	5 (0.4)	3 (60.0)	0.043^b^
Extracorporeal membrane oxygenation (ECMO)	0 (0.0)	0(0.0)	--
Cardiac catheterization	1 (0.1)	1 (100.0)	0.180^b^
Major surgery	0 (0.0)	0 (0.0)	--
Total thyroidectomy	3 (0.3)	1 (33.3)	0.449^b^

P-values were derived from univariable analyses. ^a^Chi-square test; ^b^Fisher’s Exact test; ^c^Overall comparison

The multivariable logistic regression analysis identified several independent predictors of in-hospital mortality among patients with thyroid storm. Advanced age was associated with a modest but statistically significant increase in the risk of death (adjusted odds ratio [aOR] 1.01 per year; 95% CI 1.00–1.02; p = 0.015). Male patients exhibited a 64% higher risk of mortality compared to female patients (aOR 1.64; 95% CI 1.16–2.31; p = 0.005). Among baseline comorbidities, chronic liver disease was the only independent predictor significantly associated with an unfavorable outcome (aOR 2.32; 95% CI 1.49–3.61; p < 0.001).

**Fig 3:**
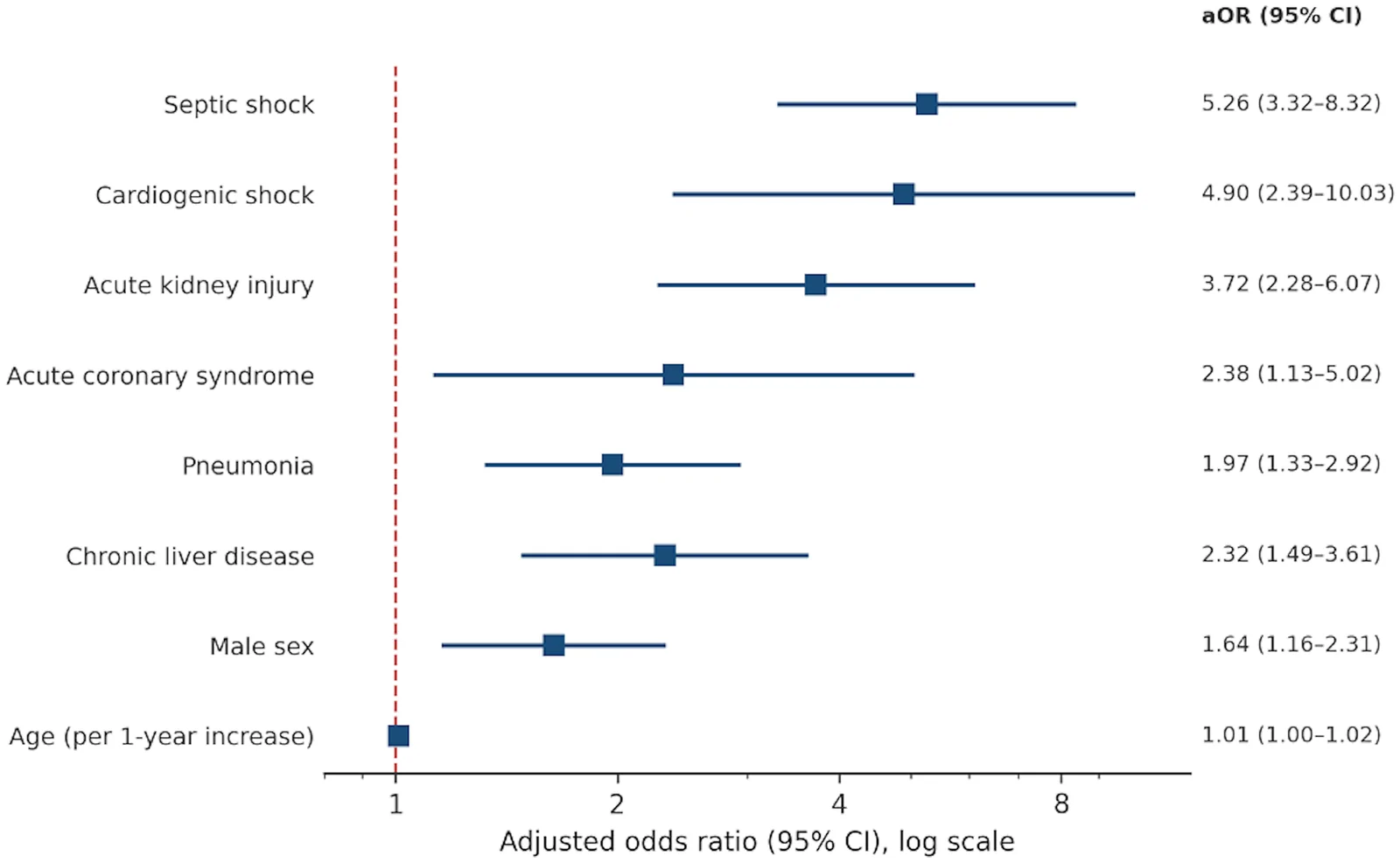
Forest plot of independent predictors of in-hospital mortality among patients with thyroid storm. Results are derived from multivariable logistic regression. Squares indicate adjusted odds ratios, and horizontal lines represent the 95% confidence intervals (log scale); the dashed vertical line marks an odds ratio of 1.

Acute inpatient complications exerted the most profound impact on survival. Septic shock and cardiogenic shock were associated with the highest risks of death (aOR 5.26; 95% CI 3.32–8.32; p < 0.001 and aOR 4.90; 95% CI 2.39–10.03; p < 0.001, respectively), followed by acute kidney injury (aOR 3.72; 95% CI 2.28–6.07; p < 0.001), acute coronary syndrome (aOR 2.38; 95% CI 1.13–5.02; p = 0.023), and pneumonia (aOR 1.97; 95% CI 1.33–2.92; p < 0.001). Cardiac arrest and ventricular arrhythmia were excluded from the multivariable model as intermediate outcomes in the causal pathway to death. Notably, chronic coronary artery disease, diabetes mellitus, and disseminated intravascular coagulation did not remain independently associated with in-hospital mortality after multivariable adjustment (Table 3 and Fig 3). The final multivariable model demonstrated good discrimination (AUC = 0.80; 95% CI 0.76–0.83) and adequate calibration (Hosmer-Lemeshow goodness-of-fit χ² = 12.955, df = 8, p = 0.113).

**Table 3:** Multivariable logistic regression analysis of factors associated with in-hospital mortality among patients with thyroid storm.

Variable	aOR	95% CI	p-value
Age (per 1-year increase)	1.01	1.00-1.02	0.015
Male sex	1.64	1.16-2.31	0.005
Chronic liver disease	2.32	1.49-3.61	< 0.001
Septic shock	5.26	3.32-8.32	< 0.001
Cardiogenic shock	4.90	2.39-10.03	< 0.001
Acute kidney injury	3.72	2.28-6.07	< 0.001
Pneumonia	1.97	1.33-2.92	< 0.001
Acute coronary syndrome	2.38	1.13-5.02	0.023

aOR, adjusted odds ratio; CI, confidence interval

### In-hospital mortality and comparative risk

**Fig 4:**
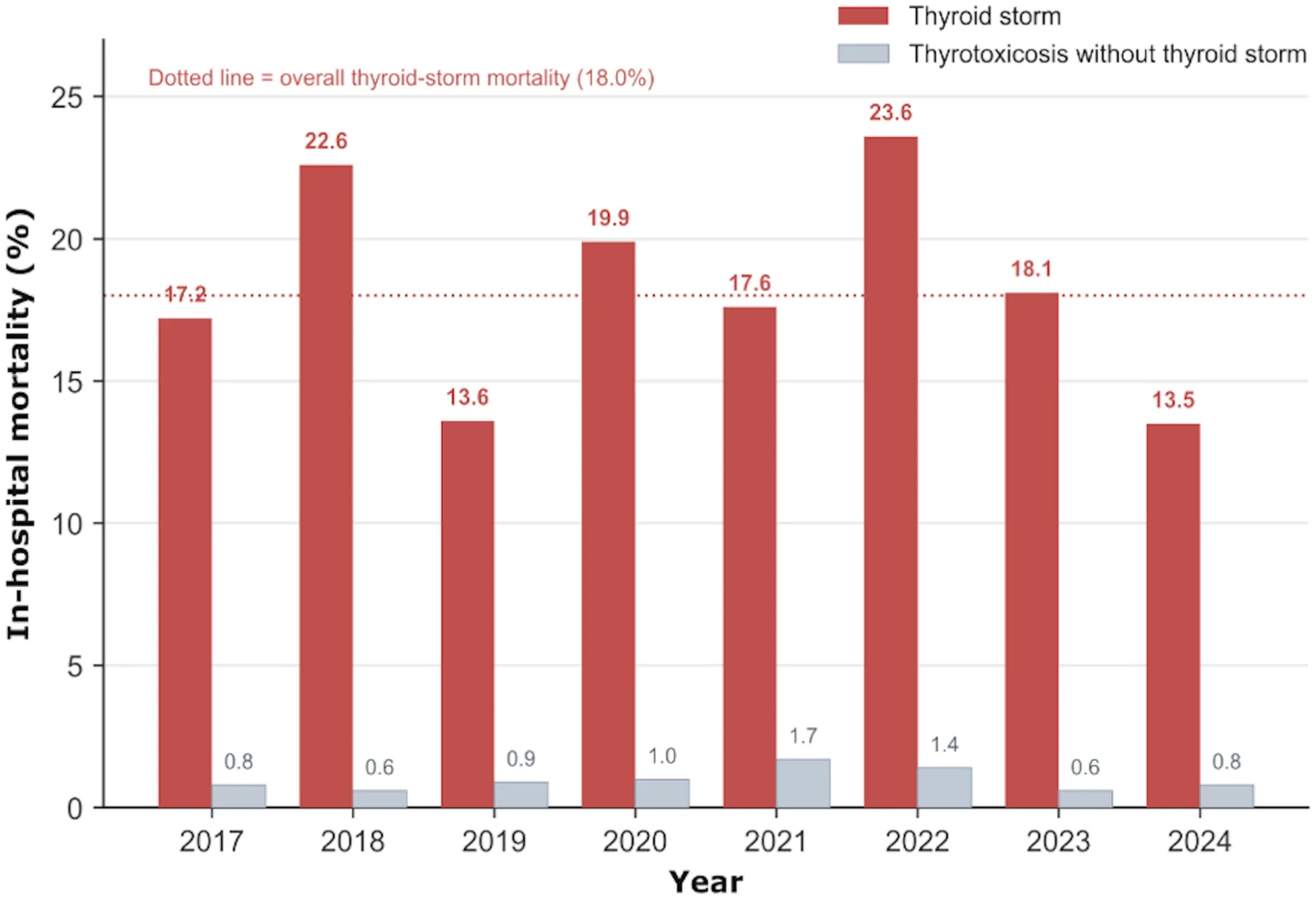
Annual in-hospital mortality among patients with and without thyroid storm in Thailand, 2017–2024. Mortality rates are presented as percentages. The red line/bars represent patients with thyroid storm, and the light line/bars represent patients without thyroid storm. The dotted horizontal line marks the overall thyroid storm mortality rate at 18.0%.

The overall in-hospital mortality rate among patients in the thyroid storm group was 18.0% (range, 13.5%–23.6%), which was markedly higher than the 1.0% mortality rate (range, 0.6%–1.7%) observed in the non-storm thyrotoxicosis group (Fig 4). In the univariable analysis, patients with thyroid storm faced a significantly higher crude risk of in-hospital death compared to those without the condition (crude odds ratio 5.98; 95% CI 4.30–8.31; p < 0.001).

After multivariable adjustment for age, sex, chronic liver disease, coronary artery disease, septic shock, cardiogenic shock, acute kidney injury, pneumonia, and acute coronary syndrome, thyroid storm remained a potent independent predictor of in-hospital mortality. The adjusted odds of death were more than eleven-fold higher in the thyroid storm cohort compared to the non-storm group (aOR 11.17; 95% CI 7.41–16.85; p < 0.001).

### Length of hospital stay (LOS)

**Fig 5:**
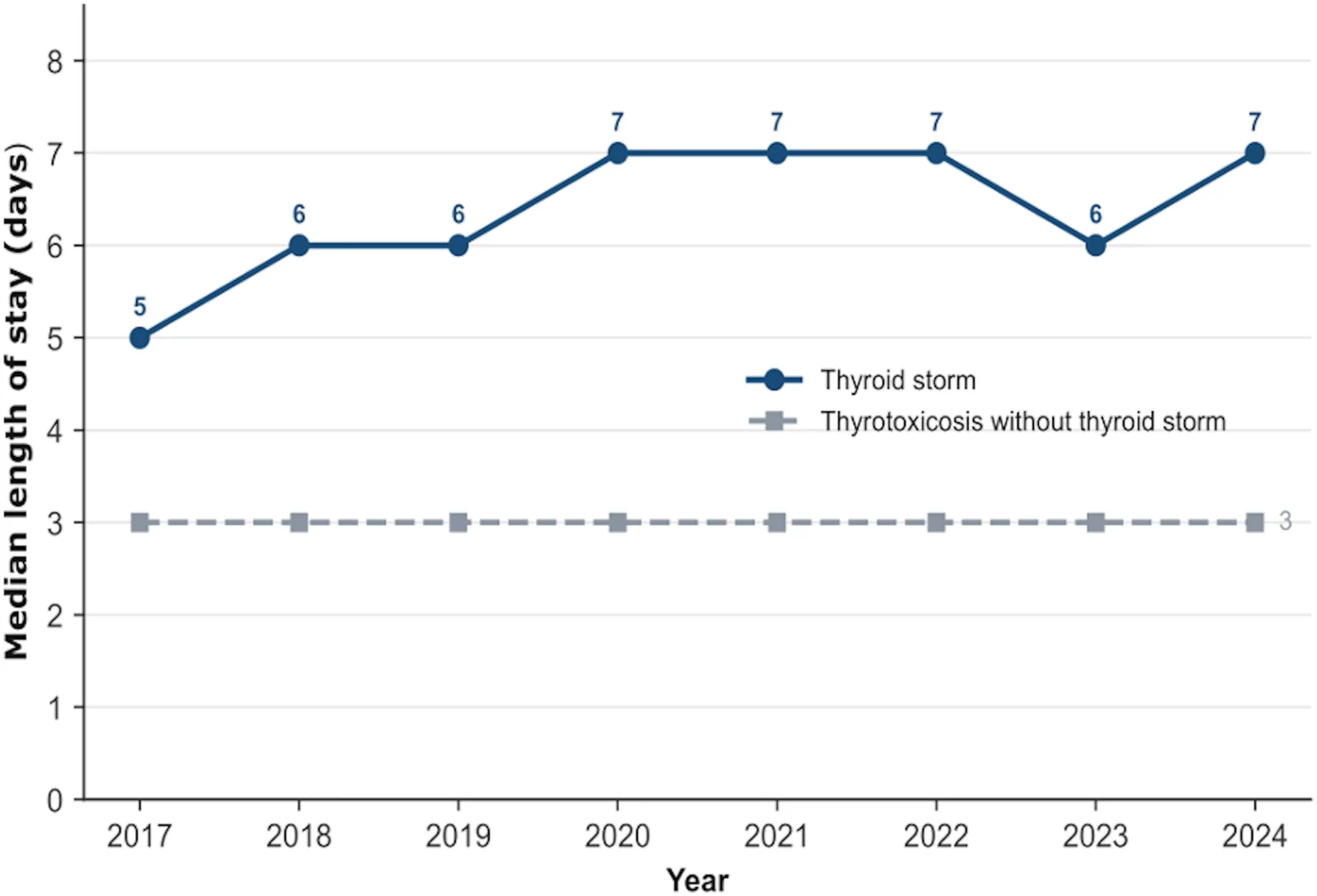
Trends in median length of hospital stay among patients admitted with thyrotoxicosis in Thailand, 2017–2024. Median length of stay is presented in days. The solid line represents patients with thyroid storm, and the dashed line represents patients without thyroid storm.

The median LOS for patients with thyroid storm was 6 days (P25–P75, 4–10), twofold longer than the 3 days (P25–P75, 2–5) observed in non-storm patients (Fig 5). In multivariable analysis, pneumonia was the strongest independent predictor of prolonged LOS (aOR 3.88; 95% CI 2.93–5.17; p < 0.001), followed by acute kidney injury (aOR 1.63; 95% CI 1.20–2.20; p = 0.002), chronic liver disease (aOR 1.69; 95% CI 1.23–2.32; p = 0.001), and older age (aOR 1.011 per year; 95% CI 1.007–1.015; p < 0.001). Acute ischemic stroke, septic shock, cardiogenic shock, and male sex were not independently associated with prolonged LOS after multivariable adjustment.

## Discussion

To the best of our knowledge, this study represents the first nationwide analysis characterizing the epidemiology and clinical outcomes of thyroid storm in Thailand. Over the eight-year study period (2017–2024), we observed a significant upward trend in the hospitalization rate of thyroid storm, rising from 0.15 to 0.32 per 100,000 population. However, this trend should be interpreted with caution, as it may partly reflect expanded NHSO network coverage over time, changes in coding practices, or disruptions in healthcare utilization during the COVID-19 pandemic (2020–2022), rather than a true increase in disease incidence. The average annual incidence of 0.22 cases per 100,000 person-years is comparable to data reported in Japan (2004–2008) but remains substantially lower than the incidence of 0.57–0.76 per 100,000 person-years reported in the United States. [2,6] This geographical disparity may be partially attributed to the application of different diagnostic criteria. While the Burch-Wartofsky Point Scale (BWPS), known for its high sensitivity, is predominantly used in the United States, clinicians in Thailand and Japan frequently apply the Japan Thyroid Association (JTA) criteria or a combination of both. [6,9] Furthermore, our finding that thyroid storm accounted for 26.2% of principal thyrotoxicosis hospitalizations, a figure notably higher than the 15.3%–18.3% reported in the United States, likely reflects differences in healthcare systems. [2] In Thailand’s national health insurance context, hospital admission is often reserved for severe clinical presentations, whereas milder cases of thyrotoxicosis may be managed as outpatients or within private sectors in other regions. Additionally, the cultural preference for medical therapy over definitive surgical intervention in Thailand may contribute to a higher prevalence of severe, poorly controlled thyrotoxicosis requiring acute care.

The in-hospital mortality rate of 18.0% observed in our cohort is strikingly higher than the 1.2%–11.3% reported in the United States, Germany, and Japan, [2,4,5,8] yet remains consistent with findings from Singapore. [7] A critical factor contributing to this higher mortality may be the limited access to advanced therapeutic interventions. For instance, therapeutic plasma exchange, a potentially life-saving modality for patients with severe liver injury or those refractory to standard medical therapy, is currently not covered by national health insurance schemes in Thailand. Although only six patients (0.5%) received therapeutic plasma exchange in this cohort, this likely represents a substantial underutilization given the severity of illness observed, and whether broader insurance coverage would improve survival remains an important hypothesis for future prospective study.

Our multivariable analysis identified male sex, advanced age, chronic liver disease, septic shock, cardiogenic shock, acute kidney injury, pneumonia, and acute coronary syndrome as independent predictors of in-hospital mortality. Cardiac arrest and ventricular arrhythmia were excluded as intermediate outcomes to avoid over-adjustment bias. The prominence of acute kidney injury underscores the importance of renal surveillance, given these patients’ susceptibility to hemodynamic instability and multi-organ failure. Proactive screening for infectious and cardiovascular complications, particularly pneumonia, which may present without overt respiratory symptoms in elderly patients, is therefore essential. [5,8]

Atrial arrhythmia and heart failure were the most prevalent comorbidities, consistent with the known cardiovascular sequelae of thyrotoxicosis. [10] Pneumonia was the third most frequent comorbidity, suggesting it may be a primary precipitating factor or a secondary complication. Noncompliance with antithyroid medication could not be identified due to the absence of specific diagnostic codes. The low prevalence of altered mental status and adrenal insufficiency likely reflects under-coding, as both are core diagnostic components of BWPS and JTA criteria. [1,6,9] A targeted approach focusing on patients with suggestive clinical features or refractory shock may therefore be more appropriate than routine cortisol measurement.

This study has several limitations inherent to administrative claims data. First, the retrospective nature may involve misclassification due to coding inaccuracies, and because thyroid storm was identified solely by the ICD-10 code E05.5 assigned by the treating physician, the validity of this code for identifying thyroid storm in the Thai NHSO system has not been formally validated against clinical diagnostic criteria such as the BWPS or JTA scores, so residual misclassification cannot be excluded.. Second, the absence of granular laboratory data precluded formal application of BWPS or JTA severity scores, and the observed low prevalence of altered mental status and adrenal insufficiency likely reflects systematic under-coding rather than true clinical absence. Third, information regarding specific medications, including antithyroid drugs, beta-blockers, and corticosteroids, was unavailable, preventing assessment of treatment-related effects on outcomes. Fourth, because each hospitalization was treated as an independent observation, patients with multiple admissions during the study period may have contributed more than once, potentially influencing incidence estimates. Fifth, ICU admission rates and duration of ICU stay were not available in the NHSO database, precluding detailed analysis of critical care utilization.

## Conclusion

This nationwide study provides the first comprehensive epidemiological analysis of thyroid storm in Thailand. The incidence is rising, with a high in-hospital mortality of 18.0%. Mortality is independently driven by advanced age, male sex, chronic liver disease, and acute multi-organ complications, particularly septic shock, cardiogenic shock, and acute kidney injury, while prolonged hospitalization is largely driven by pneumonia, acute kidney injury, and chronic liver disease. Early recognition and aggressive management of precipitating factors, particularly pneumonia, are essential to improve survival. Policymakers should consider expanding insurance coverage for advanced therapies, such as therapeutic plasma exchange, to mitigate the substantial clinical burden of this endocrine emergency.

## Supporting information

S1 Table ICD codes used to define study groups, comorbidities, clinical complications, and procedures. (DOCX)

S2 Table Collinearity statistics for variables entered into the multivariable logistic regression model for in-hospital mortality. (DOCX)

## References

[1] Burch HB, Wartofsky L. Life-threatening thyrotoxicosis. Thyroid storm. Endocrinol Metab Clin North Am. 1993;22(2):263–77. doi: 10.1016/s0889-8529(18)30165-8 8325286

[2] Galindo RJ, Hurtado CR, Pasquel FJ, García Tome R, Peng L, Umpierrez GE. National trends in incidence, mortality, and clinical outcomes of patients hospitalized for thyrotoxicosis with and without thyroid storm in the United States, 2004–2013. Thyroid. 2019;29: 36–43. 30382003 10.1089/thy.2018.0275 PMC6916241

[3] Kornelius E, Chang K-L, Yang Y-S, Huang J-Y, Ku M-S, Lee K-Y, et al. Epidemiology and factors associated with mortality of thyroid storm in Taiwan: a nationwide population-based study. Intern Emerg Med. 2021;16(3):601–7. doi: 10.1007/s11739-020-02445-6 32676839

[4] Ono Y, Ono S, Yasunaga H, Matsui H, Fushimi K, Tanaka Y. Factors Associated With Mortality of Thyroid Storm: Analysis Using a National Inpatient Database in Japan. Medicine (Baltimore). 2016;95(7):e2848. doi: 10.1097/MD.0000000000002848 26886648 PMC4998648

[5] Angell TE, Lechner MG, Nguyen CT, Salvato VL, Nicoloff JT, LoPresti JS. Clinical features and hospital outcomes in thyroid storm: a retrospective cohort study. J Clin Endocrinol Metab. 2015;100(2):451–9. doi: 10.1210/jc.2014-2850 25343237

[6] Akamizu T, Satoh T, Isozaki O, Suzuki A, Wakino S, Iburi T, et al. Diagnostic criteria, clinical features, and incidence of thyroid storm based on nationwide surveys. Thyroid. 2012;22(7):661–79. doi: 10.1089/thy.2011.0334 22690898 PMC3387770

[7] Swee DS, Chng CL, Lim A. Clinical characteristics and outcome of thyroid storm: a case series and review of neuropsychiatric derangements in thyrotoxicosis. Endocr Pract. 2015;21(2):182–9. doi: 10.4158/EP14023.OR 25370315

[8] Thiyagarajan A, Platzbecker K, Ittermann T, Völzke H, Haug U. Estimating Incidence and Case Fatality of Thyroid Storm in Germany Between 2007 and 2017: A Claims Data Analysis. Thyroid. 2022;32(11):1307–15. doi: 10.1089/thy.2022.0096 36006371

[9] Satoh T, Isozaki O, Suzuki A, Wakino S, Iburi T, Tsuboi K, et al. 2016 guidelines for the management of thyroid storm from the Japan Thyroid Association and Japan Endocrine Society. Endocrine Journal. 2016;63:1025–64. 27746415 10.1507/endocrj.EJ16-0336

[10] Kostopoulos G, Effraimidis G. Epidemiology, prognosis, and challenges in the management of hyperthyroidism-related atrial fibrillation. Eur Thyroid J. 2024;13(2):e230254. doi: 10.1530/ETJ-23-0254 38377675 PMC11046323

