# Supplementary material for: Epidemiology and mortality rate of thyroid storm in Thailand: Analysis using a national in-patient database
Source: PLoS One. 2026 Aug 27;21(8):e0357142. doi: 10.1371/journal.pone.0357142 (PMC13521574; doi:10.1371/journal.pone.0357142)
Supplement: Supplementary file 1 [file pone.0357142.s001.docx]

**S1 Table. ICD codes used to define study groups, comorbidities, clinical complications, and procedures.**

| **Variable** | **Coding system** | **Code(s)** |
| --- | --- | --- |
| **Study groups** |  |  |
| Thyroid crisis or storm | ICD-10 | E05.5 |
| Thyrotoxicosis without thyroid storm | ICD-10 | E05.0, E05.1, E05.2, E05.3, E05.4, E05.8, E05.9, E06.0, E06.1, E06.2, E06.3, E06.4, E06.5, E06.9, O90.5 |
| **Etiologies of thyrotoxicosis** |  |  |
| Thyrotoxicosis with diffuse goiter (Graves’ disease) | ICD-10 | E05.0 |
| Thyrotoxicosis with toxic single thyroid nodule | ICD-10 | E05.1 |
| Thyrotoxicosis with toxic multinodular goiter | ICD-10 | E05.2 |
| Thyrotoxicosis from ectopic thyroid tissue | ICD-10 | E05.3 |
| Thyrotoxicosis factitia | ICD-10 | E05.4 |
| Other thyrotoxicosis | ICD-10 | E05.8 |
| Thyrotoxicosis, unspecified | ICD-10 | E05.9 |
| Acute thyroiditis | ICD-10 | E06.0 |
| Subacute thyroiditis | ICD-10 | E06.1 |
| Chronic thyroiditis with transient thyrotoxicosis | ICD-10 | E06.2 |
| Autoimmune thyroiditis | ICD-10 | E06.3 |
| Drug-induced thyroiditis | ICD-10 | E06.4 |
| Other chronic thyroiditis | ICD-10 | E06.5 |
| Thyroiditis, unspecified | ICD-10 | E06.9 |
| Postpartum thyroiditis | ICD-10 | O90.5 |
| **Comorbidities & clinical factors** |  |  |
| **Baseline comorbidities** |  |  |
| Coronary artery disease | ICD-10 | I25.x |
| Chronic liver disease | ICD-10 | K70.x, K71.x, K72.1, K73.x–K77.x, B18.x |
| Chronic kidney disease | ICD-10 | N18.x |
| Chronic lung disease | ICD-10 | J40–J47.x |
| Diabetes mellitus | ICD-10 | E10.x, E11.x, E14.x |
| Depression | ICD-10 | F32.x-F33.x |
| Pregnancy | ICD-10 | O00.x-O99.x, Z33 |
| **Shock and organ failure** |  |  |
| Septic shock | ICD-10 | R57.2 |
| Cardiogenic shock | ICD-10 | R57.0 |
| Other shock | ICD-10 | R57.1, R57.8, R57.9 |
| Acute liver failure | ICD-10 | K72.0, K72.9 |
| Acute kidney injury | ICD-10 | N17.x |
| **Acute infections** |  |  |
| Sepsis | ICD-10 | A40.x, A41.x |
| Pneumonia | ICD-10 | J12.x, J13.x, J14.x, J15.x, J18.x |
| Intestinal infection | ICD-10 | A00.x-A09.x |
| Acute cystitis | ICD-10 | N30.x |
| Acute pyelonephritis | ICD-10 | N10, N11.x, N12 |
| **Cardiovascular complications** |  |  |
| Acute coronary syndrome | ICD-10 | I20.0, I21.x, I22.x |
| Heart failure | ICD-10 | I50.x |
| Atrial fibrillation and flutter, supraventricular tachycardia | ICD-10 | I48.x, I47.1 |
| Ventricular arrhythmia | ICD-10 | I47.2, I49.0 |
| Cardiac arrest | ICD-10 | I46.x |
| **Neurological complications** |  |  |
| Intracerebral hemorrhage | ICD-10 | I61.x |
| Ischemic stroke | ICD-10 | I63.x |
| Altered mental status | ICD-10 | R41.0, R40.x, F05.x |
| **Other acute complications** |  |  |
| Pulmonary embolism | ICD-10 | I26.x |
| Diabetic ketoacidosis | ICD-10 | E10.1, E11.1 |
| Adrenal insufficiency | ICD-10 | E27.x |
| Disseminated intravascular coagulation | ICD-10 | D65 |
| Trauma | ICD-10 | S00.x-T88.x |
| Non-compliance to medication | ICD-10 | Z91.1 |
| **Procedures** |  |  |
| Mechanical ventilation | ICD-9-CM | 96.70, 96.71, 96.72 |
| Hemodialysis | ICD-9-CM | 39.95 |
| Therapeutic plasma exchange | ICD-9-CM | 99.71 |
| Intra-aortic balloon pump | ICD-9-CM | 37.61 |
| Extracorporeal membrane oxygenation | ICD-9-CM | 39.65 |
| Cardiac catheterization | ICD-9-CM | 37.21, 37.22, 37.23 |
| Major surgery | ICD-9-CM | 35.xx - 39.xx |
| Total thyroidectomy | ICD-9-CM | 06.4 |

ICD-10: International Classification of Diseases, 100th Revision; ICD-9-CM: International Classification of Diseases, 9th Revision, Clinical Modification.
