# Supplementary material for: Epidemiology and mortality rate of thyroid storm in Thailand: Analysis using a national in-patient database
Source: PLoS One. 2026 Aug 27;21(8):e0357142. doi: 10.1371/journal.pone.0357142 (PMC13521574; doi:10.1371/journal.pone.0357142)
Supplement: Supplementary file 2 [file pone.0357142.s002.docx]

**S2 Table. Collinearity statistics for variables entered into the multivariable logistic regression model for in-hospital mortality.**

| **Variable** | **Tolerance** | **VIF** |
| --- | --- | --- |
| ***Demographics*** | | |
| Age (per 1-year increase) | 0.863 | 1.159 |
| Male sex | 0.930 | 1.075 |
| ***Baseline Comorbidities*** | | |
| Chronic liver disease | 0.922 | 1.084 |
| Coronary artery disease | 0.958 | 1.044 |
| Diabetes mellitus | 0.912 | 1.096 |
| Chronic kidney disease | 0.917 | 1.090 |
| Chronic lung disease | — | — |
| ***Shock and Organ Failure*** | | |
| Septic shock | 0.785 | 1.274 |
| Cardiogenic shock | 0.886 | 1.129 |
| Acute kidney injury | 0.719 | 1.391 |
| Acute liver failure | 0.858 | 1.165 |
| ***Cardiovascular Complications*** | | |
| Acute coronary syndrome | 0.919 | 1.089 |
| Heart failure | 0.761 | 1.314 |
| Atrial arrhythmia | 0.781 | 1.281 |
| Ventricular arrhythmia* | 0.871 | 1.149 |
| Cardiac arrest* | 0.833 | 1.201 |
| ***Infection*** | | |
| Pneumonia | 0.800 | 1.249 |
| ***Neurological Complications*** | | |
| Acute ischemic stroke | 0.941 | 1.063 |
| ***Procedures*** | | |
| Mechanical ventilation | 0.609 | 1.643 |
| Hemodialysis | 0.865 | 1.156 |
| ***Other*** | | |
| Disseminated intravascular coagulation | 0.906 | 1.103 |
| Adrenal insufficiency | 0.975 | 1.025 |

VIF, variance inflation factor.

All VIF values were below 5.0, indicating no significant multicollinearity among predictor variables.

Variables listed represent all candidates assessed for multicollinearity prior to final model selection; not all variables were retained in the final multivariable model.

- Cardiac arrest and ventricular arrhythmia were excluded from the final multivariable model as they were considered intermediate outcomes in the causal pathway to death rather than independent predictors of mortality.

— Insufficient cell count for reliable VIF estimation; variable excluded from model.
